# Recent strides toward transforming lignin into plastics and aqueous electrolytes for flow batteries

**DOI:** 10.1016/j.isci.2024.109418

**Published:** 2024-03-04

**Authors:** Omar Y. Abdelaziz, Mariona Battestini Vives, Smita V. Mankar, Niklas Warlin, Tran Tam Nguyen, Baozhong Zhang, Christian P. Hulteberg, Amirreza Khataee

**Affiliations:** 1 Department of Chemical Engineering, King Fahd University of Petroleum & Minerals, Dhahran 31261, Saudi Arabia; 2 Interdisciplinary Research Center for Refining & Advanced Chemicals, King Fahd University of Petroleum & Minerals, Dhahran 31261, Saudi Arabia; 3 Division of Chemical Engineering, Department of Process and Life Science Engineering, Lund University, SE-221 00 Lund, Sweden; 4 Centre for Analysis and Synthesis, Department of Chemistry, Lund University, SE-221 00 Lund, Sweden; 5 Department of Chemistry, Stanford University, Stanford, CA 94306, USA; 6 Division of Applied Electrochemistry, Department of Chemical Engineering, KTH Royal Institute of Technology, SE-100 44 Stockholm, Sweden

**Keywords:** Biomass, Biotechnology, Chemical engineering, Engineering

## Abstract

Lignin is an abundant polyaromatic polymer with a wide range of potential future uses. However, the conversion of lignin into valuable products comes at a cost, and medium- to high-value applications are thus appropriate. Two examples of these are polymers (e.g., as fibers, plasticizers, or additives) and flow batteries (e.g., as redox species). Both of these areas would benefit from lignin-derived molecules with potentially low molecular weight and high (electro)chemical functionality. A promising route to obtain these molecules is oxidative lignin depolymerization, as it enables the formation of targeted compounds with multiple functionalities. An application with high potential in the production of plastics is the synthesis of new sustainable polymers. Employing organic molecules, such as quinones and heterocycles, would constitute an important step toward the sustainability of aqueous flow batteries, and lignin and its derivatives are emerging as redox species, mainly due to their low cost and renewability.

## Graphical abstract

**Figure:**
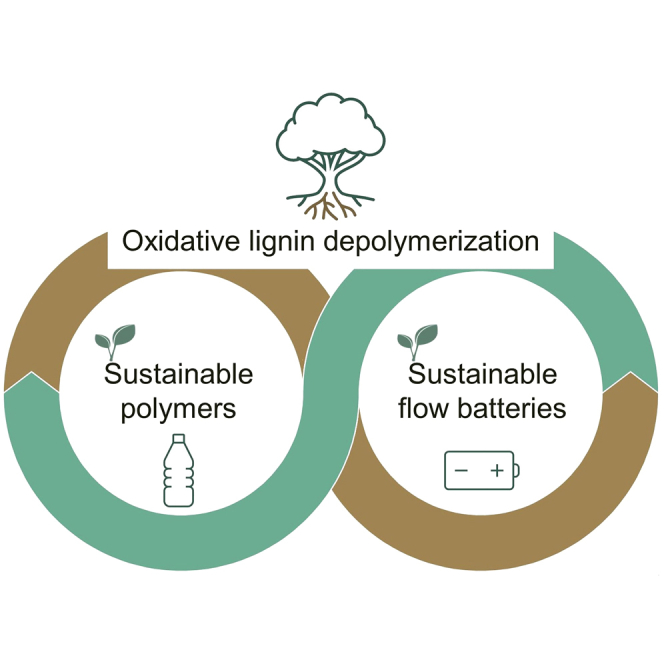

## Introduction

Lignin is a renewable biopolymer and one of the three main components of wood. Currently, the annual global production of lignin from the pulp and paper industry is estimated to be 50–70 million tons.^1^^,^^2^ The primary source of lignin is black liquor, which is generated as a by-product in the pulp and paper industry. The black liquor contains about 30–45 wt % lignin and is usually burned for energy recovery.^3^ However, lignin can be extracted and used in various value-added applications, for instance, as a sustainable resource for various chemicals, as chemical building blocks and polymers, or as an electrode or separator in various types of battery.^4^^,^^5^

Plastics have been associated with serious environmental problems such as pollution of the oceans, the generation of microplastics, and health problems associated with the toxicity of additives or monomers. Many of these issues are closely connected to the use of fossil resources as starting materials for polymer production. Lignin has been identified as a potential bio-based carbon source for polymer production as it does not compete with food production, and is readily available at a low cost. Lignin can be used directly as a polymer, and for more information on this topic, the reader is referred to recent reviews on the use of lignin in composites/blends,^6^^,^^7^ bio-based fillers/plasticizer for plastics,^8^ and stimuli-responsive materials,^9^ and in antimicrobial and agricultural applications.^10^ However, this perspective article focuses on the use of depolymerized lignin as a starting material for the synthesis of bio-based polymers.

Nonetheless, lignin has electrochemical properties, potentially making it a sustainable material for electrochemical devices. Lignin can be used as a redox species for batteries, the best example being the redox flow battery (RFB). The most commonly used redox species for RFBs are metal-based and extraction requires costly mining processes. In addition, some metals, such as vanadium, are scarce (and are included on the list of critical materials), toxic, and very expensive.^11^ Although organic redox species such as quinones have been introduced in recent years as a cost-effective and abundant alternative, their large-scale production is costly and challenging.^12^^,^^13^ Starting materials derived from petroleum or coal, such as aromatic hydrocarbons, are chemically modified and transformed into quinones through steps such as oxidation, condensation, and cyclization. Therefore, using lignin as a redox species would be a significant step toward the production of sustainable electrolytes for RFBs, as it is a bio-based, renewable, and low-cost material.

## Lignin transformation

**Figure 1:**
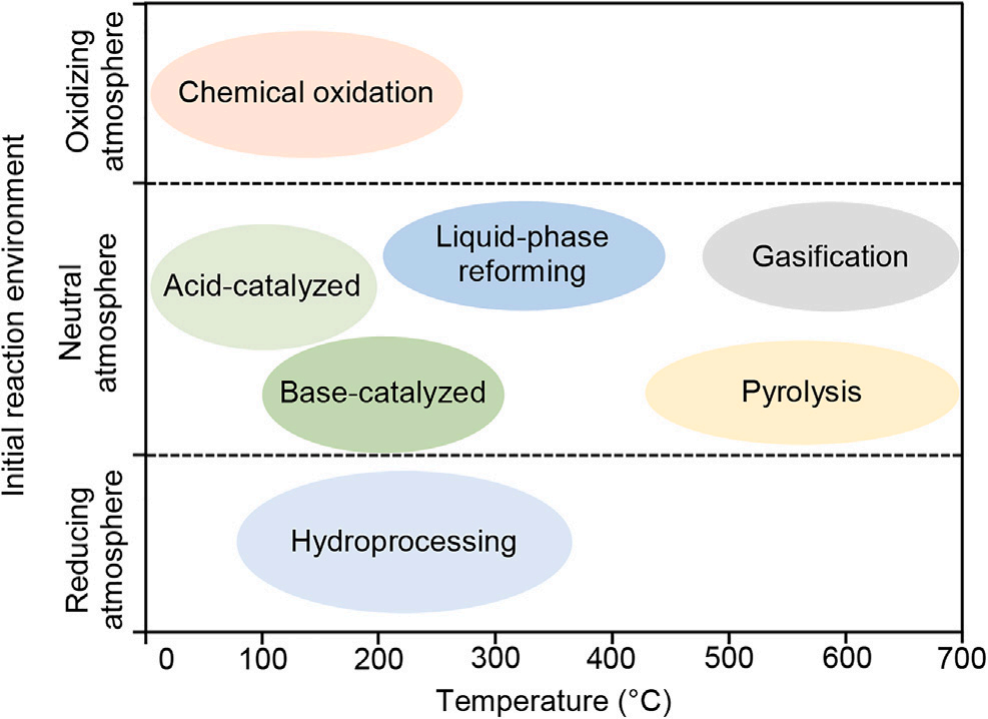
An overview of thermochemical technologies for lignin transformation The typical temperature ranges for the different technologies can be seen on the abscissa.^14^^,^^16^.

In this article, lignin transformation means the conversion of the lignin macromolecule into low-molecular-weight compounds. This can be achieved by thermochemical or biological means. Thermochemical transformation can be classified as acid- or base-catalyzed depolymerization (hydrothermal treatment), chemical oxidation (oxidative depolymerization), hydroprocessing, liquid-phase reforming, pyrolysis, and gasification (Figure 1).^14^^,^^15^

Acid-catalyzed depolymerization is performed at 0°C to 200°C, while base-catalyzed depolymerization takes place at 100°C to 300°C. Both these kinds of depolymerization cause the linkages between the lignin units to be broken, yielding shorter segments, including monomeric phenols. Chemical oxidation is performed at temperatures ranging from 0°C to 250°C, and favors the production of aromatic alcohols, aldehydes, and acids. Hydroprocessing involves thermal reduction in the presence of a hydrogen source at temperatures ranging from 100°C to 350°C. This technique is used to produce phenols, benzene, toluene, xylene, and also alkane fuels, from lignin. Liquid-phase reforming, which is typically performed at 250°C to 400°C, is used to produce hydrogen and other light gases from lignin. The pyrolysis of lignin is typically carried out at 450°C to 700°C, and produces a liquid product known as bio-oil. Gasification is the process used to produce synthesis gas (CO and H_2_) from a range of lignin feedstocks. The lignin-degrading activity of certain bacteria and fungi has also been explored in attempts to develop biological methods of breaking down lignin with high selectivity for specific molecules.^17^^,^^18^

This perspective article focuses on oxidative depolymerization, during which, lignin is converted into polyfunctional aromatic compounds in the presence of an oxidizing agent. Oxidation includes the cleavage of the aryl ether bonds, carbon–carbon bonds, aromatic rings, and other linkages within the lignin molecule. The resulting products are phenolic aldehydes and acids, as well as aliphatic carboxylic acids, such as vanillin, syringaldehyde, 4-hydroxybenzaldehyde, and muconic acid.^14^ The most common oxidizing agents are molecular oxygen (O_2_), hydrogen peroxide (H_2_O_2_), nitrobenzene, and metal oxides. The reactions can take place in alkaline, acidic, or pH-neutral media, employing homogeneous or heterogeneous catalysts.^16^ Catalysts are mainly used during oxidative depolymerization to reduce the energy consumption, reaction time, temperature, and pressure. Catalysts also improve the monomer yields and the selectivity of the reaction.

Historically, vanillin production has been the focus of oxidative depolymerization research.^19^^,^^20^ However, researchers are now exploring other ways of valorizing lignin as society is shifting from a petroleum-based toward a more sustainable bio-based economy. Recent studies and reviews have concentrated on the electrochemical applications of lignin, such as supercapacitors and electrodes.^21^^,^^22^^,^^23^ Oxidative depolymerization has been shown to transform lignin into different quinone-like structures,^24^ which are key compounds for the electrochemical reactions that take place within a battery.^22^

Oxidation of monolignols has been suggested by Milczarek as a method of electrode preparation.^25^ It has been shown that it is possible to oxidize the syringyl (S) and guaiacyl (G) monolignols to quinone-like structures as they carry methoxy structures. Lignin-derived quinone structures have been shown to have potential in redox-flow battery applications.^26^ Most studies presented in the literature concern the conversion of lignin model compounds into benzoquinones. For example, Biannic and Bozell obtained methoxybenzoquinone and 2,6-dimethoxybenzoquinone (DMBQ) as primary products after base-catalyzed oxidation of monomeric and dimeric lignin model compounds.^27^ However, the possibility of using real lignin feedstocks has also been investigated. For example, Subbotina et al. demonstrated an oxidation reaction that produced DMBQ from high-molecular-weight lignin at a yield of 18%.^28^ They proposed cleavage of the carbon–carbon bonds, which is often difficult during oxidative depolymerization.

## Lignin-based plastics

Lignin is an abundant renewable source of aromatic structures and has therefore been used to prepare aromatic monomers and polymers. Aromatic structures are often used in polymers to improve their rigidity, which also improves the thermal and mechanical properties of the material. Furthermore, due to the relatively high oxygen content in lignin, much research has been directed toward the synthesis of monomers and polymers with high contents of aromatic structures and oxygen, e.g., polyesters, polycarbonates, and epoxies. Vanillin is the only lignin-derived molecule that has reached industrial commercialization to date. Vanillic acid is another promising molecule that can be derived from oxidative lignin depolymerization with potential for commercialization, and efforts are still underway to enable economical routes for its production.^29^ Hence, this perspective article focuses mainly on vanillin-based monomers and polymers via three common types of monomer: polyols, dicarboxylic acids (including derived diesters), and hydroxy acids (including acetylated hydroxy acids) as shown in Table 1.

**Table 1:** Vanillin-based monomers used in polymer synthesis

No.	Monomers and their derivatives	Polymer	Properties^a^	Potential application	Reference
**Polyols**					

**1**		Polycarbonate Epoxy	Polycarbonate: *T*_g_ = 99°C–106°C *T*_5_ = 336°C–372°C Epoxy: *T*_g_ (*E*″) = 100°C–123°C *T*_g_ (Tanδ) = 107°C–132°C *E*' = 2.29–3.02 GPa *T*_5_ = 337°C–363°C Lower estrogen activity than BPA	Direct replacement for BPA, adhesives	Koelewijn and Hernandez et al. ^30^^,^^31^
**2**		Polyester	*T*_g_ = 42°C–142°C *T*_d_ = 272°C–342°C	Fiber	Wu et al. ^32^
**3**		Epoxy Polyester	Recyclability Epoxy: *T*_g_ = 74°C–119°C *E*' = 1.15 GPa (neat) – 7.6 GPa (reinforced with CNF) Epoxy2: *T*_5_ = 278°C *T*_g_ = 164°C Tensile modulus = 3131 MPa Tensile strength = 85 MPa Polyester: *T*_g_ = 20°C–64°C *T*_5_ = 320°C–345°C *E*' (20°C) = 756–1991 MPa	Fiber Polyesters: textile, food packaging	Mankar, Koike, Mankar, and Subbotina et al. ^33^^,^^34^^,^^35^^,^^36^
**4**		Poly(vanillin) oxalate	Biodegradable, biocompatible	Medical devices, drug delivery	Kwon et al. ^37^
**5**		Polyesters Epoxies Polyurethanes	Polyesters: *T*_g_ = -5–139°C *E*' = 0.1–8.1 GPa *T*_5_ = 270°C–347°C Potential phenolic functionality Epoxies: *T*_g_ = 138°C–198°C *T*_α_ = 155°C–200°C *E*' = 1.7–2.4 GPa *T*_5_ = 275°C–336°C Potential flame retardancy Polyurethanes: *T*_5_ = 330°C–342°C Young’s modulus = 8.0–9.7 MPa	Polyesters: Epoxies: potential bio-based alternative for DGEBA (bisphenol A diglycidyl ether)	Llevot, Savonnet and Gang et al^38^^,^^39^^,^^40^
**6**		Epoxies	*T*_g_ = 132°C, 97°C *T*_α_ = 154°C, 106°C *E*' (30 C) = 1.2, 1.5 GPa *T*_d_ = 338°C, 361°C CY = 20%, 19%	To replace BPA-based epoxies	Fache et al. ^41^

**Dicarboxylic acids/diesters**					

**7**	**7a**: R = H, n = 2 **7b**: R = CH_3_, n = 4	Polyesters	**7a**: *T*_g_ = 55°C–69°C *T*_*m*_ = 212°C–260°C **7b**: *T*_g_ = −4.4°C–13°C *T*_*m*_ = 70.1°C *T*_5_ = 360°C–390°C *E*' = 493–581 MPa Tensile strength = 5.0–7.0 MPa Strain at break = 12.7–43.7% Young’s modulus = 66.2–99.7 MPa	Fibers	Lange and Pang et al. ^42^^,^^43^
**8**		Polyesters	*T*_g_ = −10.3 to −12.7°C *T*_*m*_ = 24.5, 48.5°C *T*_5_ = 335°C–339°C *E*' = 283 MPa Tensile strength = 4.1 MPa Strain at break = 22.8% Young’s modulus = 50 MPa	Biodegradable polyesters	Pang et al. ^43^
**9**	R = (CH_2_)_n_CH_3_; n = 0,1,2 & 3	Polyesters	*T*_g_ = 19°C–89°C *T*_5_ = 340°C–390°C Tensile strength = 0.34–15.8 MPa Elongation at break = 55–1880% Young’s modulus = 0.13–1 MPa	Packaging	Enomoto et al. ^44^

**Hydroxy acids/acetylated hydroxyl acids**					

**10**		Polyesters	*T*_*g*_ = 73°C *T*_*m*_ = 234°C Comparable characteristics to PET	Textiles	Mialon et al. ^45^
**11**	n = 0, 2, 3, 6, & -CH(CH_3_)CH_2_-	Polyesters	Comparable characteristics to commercial polyesters such as PET, PBT depending on aliphatic spacers (n)	Textiles	Mialon, Zamboulis and Gioia et al. ^46^^,^^47^^,^^48^

a *T*_g_ = glass transition temperature (measured by differential scanning calorimetry), *T*_g_(*E*″) = glass transition temperature (measured from the loss modulus curve by dynamic mechanical analysis), *T*_g_(Tanδ) = glass transition temperature (measured from the Tanδ curve by dynamic mechanical analysis), *T*_m_ = melting temperature, *T*_5_ = decomposition temperature at 5% mass loss, *T*_d_ = temperature at the maximum degradation rate, *T*_α_ = alpha transition temperature, *E*’ = storage modulus, *E*’’ = loss modulus, CY = char yield.

### Polyols

Polyols are building blocks commonly used to produce a wide variety of plastics such as polyesters, polyurethanes, polycarbonates, and epoxy resins. The chemical structure of polyols plays an essential role in the properties of the resulting polymer. Structurally rigid polyols are often designed to produce polymers with high glass transition temperatures and good mechanical properties. Lignin is especially suitable as a starting material for the production of rigid aromatic polyol monomers due to its high aromatic content, as well as its relatively high oxygen content. Since vanillin contains a phenolic hydroxyl group, significant efforts have been made to convert vanillin into different bisphenolic structures, often with the intention of replacing the commercially available monomer bisphenol A (BPA). About 8 million tons BPA is currently produced per year, and is used primarily in polycarbonates and epoxy resins.^49^ However, since BPA has been identified as an endocrine disruptor, many non-toxic bio-based alternatives have been developed.

**Scheme 1:**
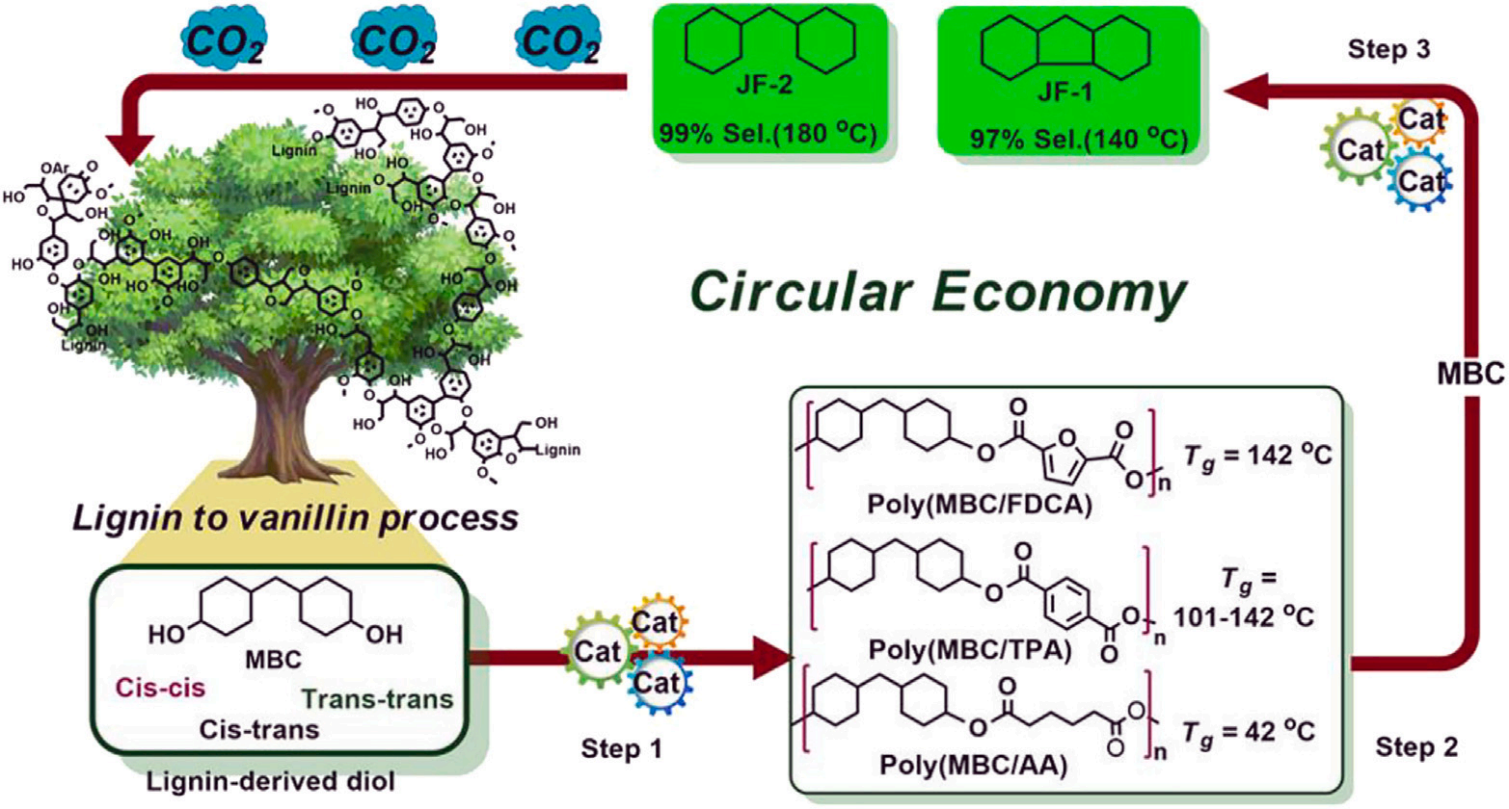
Overview of the synthesis and depolymerization of bio-based polyesters from a bicyclic lignin-derived diol (MBC, 4,4′-methylenebiscyclohexanol; FDCA, 2,5-furandicarboxylic acid; TPA, terephthalic acid; AA, adipic acid; JF-1, perhydrofluorene; JF-2, dicyclohexylmethane). Reproduced from ref. ^32^, under the terms of the Creative Commons Attribution 4.0 International License (https://creativecommons.org/licenses/by/4.0/).

Bisguaiacol (**1a**), synthesized from vanillyl alcohol and guaiacol, is structurally similar to BPA, and it has attracted significant attention as a potential replacement.^30^^,^^31^ Interestingly, the endocrine effect of **1a** and the activity toward the estrogen receptors is significantly lower than that of BPA.^30^^,^^50^ However, a more comprehensive analysis of **1a** is required to confirm that it does not interact with other hormonal receptors. **1a** has been used to prepare polycarbonates with slightly lower glass transition temperatures and thermal stability than the commonly produced BPA-based polycarbonates.^30^ **1a** has also been epoxidized (**1b**) and used to prepare epoxy resins, which also had slightly lower glass transition temperatures and thermal stability than BPA-based materials.^31^ Recently, **1a** was hydrogenated and de-methoxylated using Raney nickel to prepare a cyclic diol (**2**) for the synthesis of polyesters with a wide range of glass transition temperatures (42°C–142°C) for potential fiber applications (Scheme 1).^32^

**Scheme 2:**
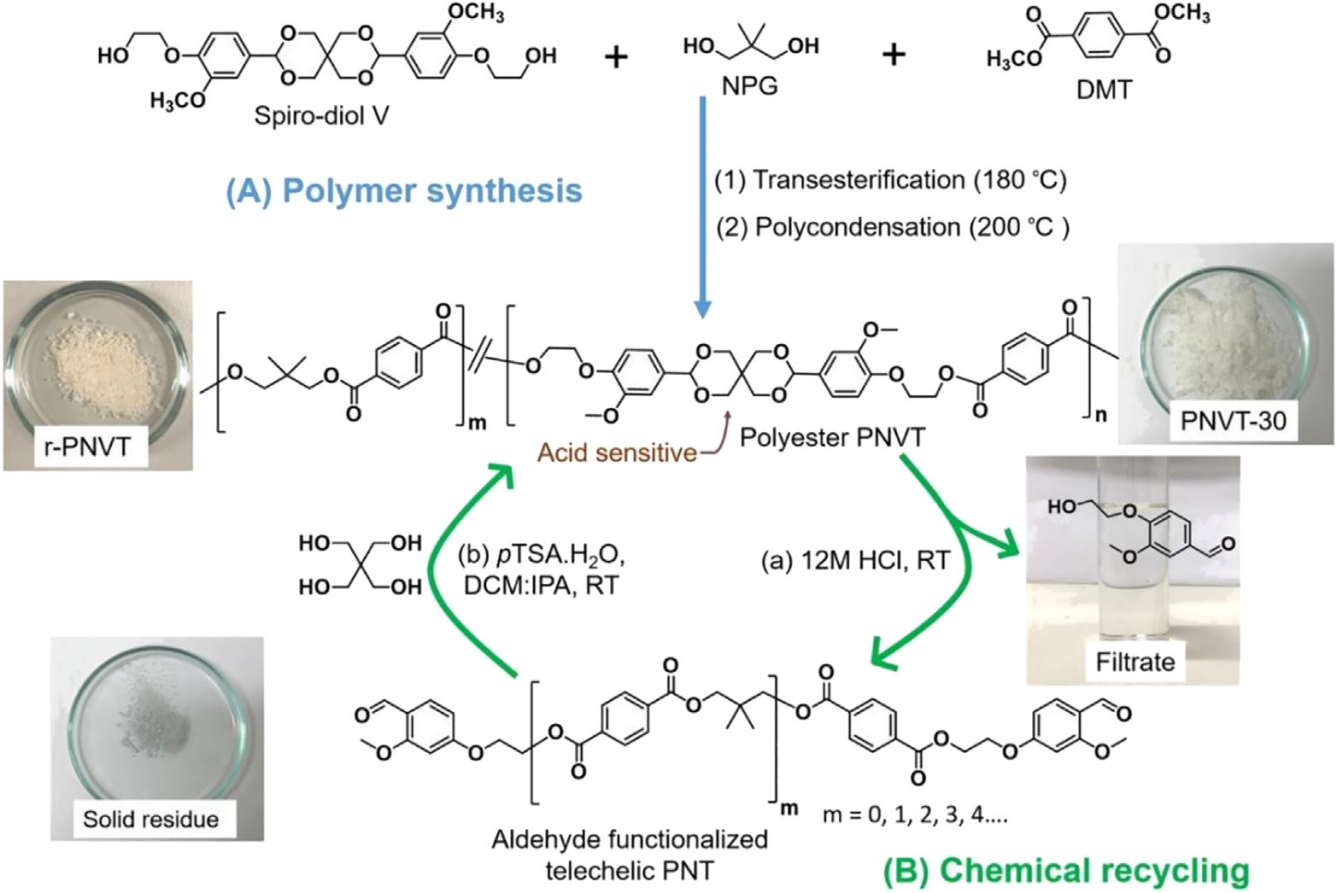
Synthesis and chemical recycling of vanillin-based polyesters with spirocyclic acetals (A) Polymer synthesis and (B) chemical recycling of copolyesters with spirocyclic acetal structures. (NPG, neopentyl glycol; DMT, dimethyl terephthalate; *p*TSA·H_2_O, *p*-toluenesulfonic acid monohydrate; DCM, dichloromethane; IPA, isopropanol; RT, room temperature; PNT, poly(neopentyl terephthalate)). PNVT is a copolyester of the vanillin-based monomer V, NPG, and DMT. Reproduced from ref. ^35^, under the terms of the Creative Commons Attribution 4.0 International License (https://creativecommons.org/licenses/by/4.0/).

Another widely studied vanillin-based bisphenol is **3a**, which can be made from bio-based pentaerythritol and vanillin.^33^^,^^51^^,^^52^ One aspect that makes this molecule particularly interesting is its rigid spirocyclic acetal structure, which can be selectively hydrolyzed when exposed to acidic conditions. This feature has been used as a strategy for facilitating the chemical recycling of polymers such as epoxy resins and polyesters (Scheme 2).^35^^,^^36^ Apart from its advantageous material properties, recyclability and end-of-life should be given greater consideration when designing novel bio-based monomers/polymers.^53^ Modification of **3a** with ethylene carbonate produces the rigid primary diol (**3c**), which has been used for polyester synthesis.^33^ This diol has a remarkably low carbon footprint, smaller than that of BPA and bio-based propane diol. Furthermore, a series of polyesters from **3c** with enhanced *T*_g_ has been prepared by bulk polycondensation. However, the spirocyclic structure exhibited relatively low thermal stability during high-temperature polycondensation, indicating that the polymerization parameters may require optimization.

Another acetal structure (**4)** has also been prepared by reacting vanillin with 2-(hydroxymethyl)-2-methylpropane1,3-diol.^37^ This monomer was reacted with oxalyl chloride to prepare acid-sensitive polyesters. These polymers are hydrolyzed at physiological pH, releasing vanillin, which acts as an antioxidant and reduces oxidative stress and inflammation.

Two vanillin molecules can also be coupled directly to yield bis-vanillin.^40^^,^^54^ Bis-vanillin has been studied extensively and can be reduced to primary alcohols (**5a**) (which can be used for polyester synthesis after methoxylating the phenol), converted into epoxies (**5b**), or used as a chain extender for polyurethane synthesis (**5c**).^38^^,^^39^^,^^40^ The possibility of using unreacted phenols or aldehydes for post-polymerization modification of the polymer is particularly interesting for these types of structure, as it could be used to endow the polymer with desirable properties.

Methoxyhydroquinone and other monoaromatic diols (**6a-b**) could be used to prepare diglycidyl ethers for bio-based epoxies.^41^ The glass transition temperature of the obtained epoxy polymers ranged from 97°C to 132°C, depending on the precursor (methoxyhydroquinone or vanillyl alcohol).^41^ These resins exhibited a relatively high char yield due to the high content of aromatic carbon in their chemical structure, which may indicate flame-retardant properties.

### Dicarboxylic acids

Dicarboxylic acids (including their derivatives, such as dicarboxylate esters) constitute an important group of monomers that can be used to produce polyesters and polyamides. The most common dicarboxylic acid on the market is fossil-based terephthalic acid (TPA), an aromatic building block with an estimated market volume of 82 million metric tons in 2021, which is expected to increase to more than 100 million tons by 2028.^55^ Dicarboxylic acids such as TPA can be esterified with methanol to yield their corresponding methyl esters (e.g., dimethyl terephthalate [DMT]), which can be used to prepare the same type of polymers, but are generally more convenient to work with due to their lower melting temperatures. Nevertheless, TPA is still preferred by industry due to its lower cost. Since TPA is exclusively produced from fossil resources in industry, extensive research efforts have been devoted to finding suitable alternative bio-based dicarboxylic acids (or diesters). In the early 1980s, a diacid (**7a**) was synthesized by bridging the phenolic groups of two vanillic acid molecules with ethylene glycol.^42^ **7a** was then polymerized with ethylene glycol to obtain semicrystalline polyesters with *T*_g_ ∼ 55°C–69°C and *T*_m_ >200°C. These polymers may be suitable for fiber applications due to their high crystallinity. More recently, a similar diester monomer, **7b,** was reported and used in polycondensation with long-chain fatty diols to produce amorphous or semicrystalline polyesters with relatively low *T*_g_ (and low *T*_m_ if there is crystallinity).^43^ Another diester monomer (**8**) with one aromatic and one aliphatic ester bond was made from methyl vanillate.^43^ Polyesters produced using this monomer had relatively low melting points and may be suitable for biodegradable packaging or fiber applications.

Another interesting example is methylated dimethyl divanillate (**9**), which is a diester monomer suitable for polyester synthesis.^44^ This monomer can be prepared by dimerization of vanillic acid, a potential product from oxidative lignin depolymerization or vanillin oxidation.^56^ The polyesters synthesized from this monomer (**9)** were completely amorphous with excellent thermal stability and tunable *T*_g_ ranging from 19°C to 89°C.^44^ These polymers were successfully cast into films, indicating that they might be suitable for packaging applications.

### Hydroxy acids

Lignin-derived monoaromatic building blocks with various functional groups (e.g., aldehyde, phenolic alcohol, carboxylic acids, and carboxylate esters) can be used to fabricate various AB-type monomers (e.g., with one OH and one COOH group, or their derivatives). These monomers can be self-polymerized to prepare polyesters that can, to some extent, mimic the structures and properties of polyethylene terephthalate (PET) and polybutylene terephthalate (PBT).^57^ One of the main advantages of AB monomers is that the requirement for stoichiometric balance in conventional step-growth polymerization is in principle fulfilled, which may facilitate the synthesis of high-molecular-weight polymers. However, due to the possibility of side reactions of the functional groups, stoichiometric imbalance could still be a problem. Furthermore, the cost of unsymmetrical AB monomers may be higher than that of symmetrical AABB monomers (e.g., TPA, DMT, etc.), which could also present a challenge in upscaling investigations.

An interesting AB monomer (**10**) has been reported by reacting dihydroferulic acid with acetic anhydride.^45^ This monomer self-polymerized to yield a polyester called poly(dihydroferulic acid), which could be a potential bio-based alternative to PET, with *T*_*g*_ = 73°C and *T*_*m*_ = 234°C. The reactivity of the phenolic hydroxyl group is increased by an acetylation reaction of the hydroxy group which takes place in tandem with the Perkin reactions of the aldehyde group. Interestingly, dihydroferulic acid can be prepared from ferulic acid, which can be directly derived from lignin.^58^

Another strategy for utilizing monoaromatics from lignin depolymerization is to improve the reactivity of phenols by alkylation to yield more reactive alcohol, for example, by reaction with halide alkanols to introduce more nucleophilic primary alcohol. For instance, several AB-type hydroxy acids have been synthesized from vanillic acid, syringic acid, and 4-hydroxy benzoic acid using this method (**11**).^46^ The polyesters prepared from these monomers showed comparable properties to those of PET and PBT. Another PET-analogous polyester, poly(ethylene vanillate) (PEV), was prepared from a vanillic-acid-based monomer, which also exhibited comparable thermal properties to PET (*T*_g_ = 80°C, and *T*_m_ = 251°C–261°C).^47^ The elastic modulus of PEV was found to be 1506 N/mm^2^ and the indentation hardness 178 N/mm^2^, which are in the same range as those of amorphous and annealed PET and polypropylene.

In principle, the use of AB-type monomers in copolymerization with other AB-type monomers (e.g., caprolactone, lactide, etc.) will allow fine-tuning of the properties of the resulting polymers, as has been occasionally reported.^48^^,^^59^

## Lignin-based electrolytes for flow batteries

Due to the non-renewable nature of fossil fuels and the environmental issues associated with their use, finding environmentally friendly energy sources is a necessity. Renewable energy sources such as solar and wind power are the best alternatives to fossil fuels, but they suffer from discontinuity and intermittence. Advanced energy storage technology is needed to overcome these problems. Among the available alternatives, the RFB is an excellent candidate. A competitive advantage that distinguishes RFBs from other batteries is the independent scale of power and energy. The latter makes RFBs suitable for long-duration energy storage applications on the medium to large scale (kWh to MWh). Also, unlike lithium-ion batteries, the electrodes do not experience failures such as phase transformations or changes in morphology. Furthermore, RFBs are generally not damaged by overcharging or high depth of discharge.

**Figure 2:**
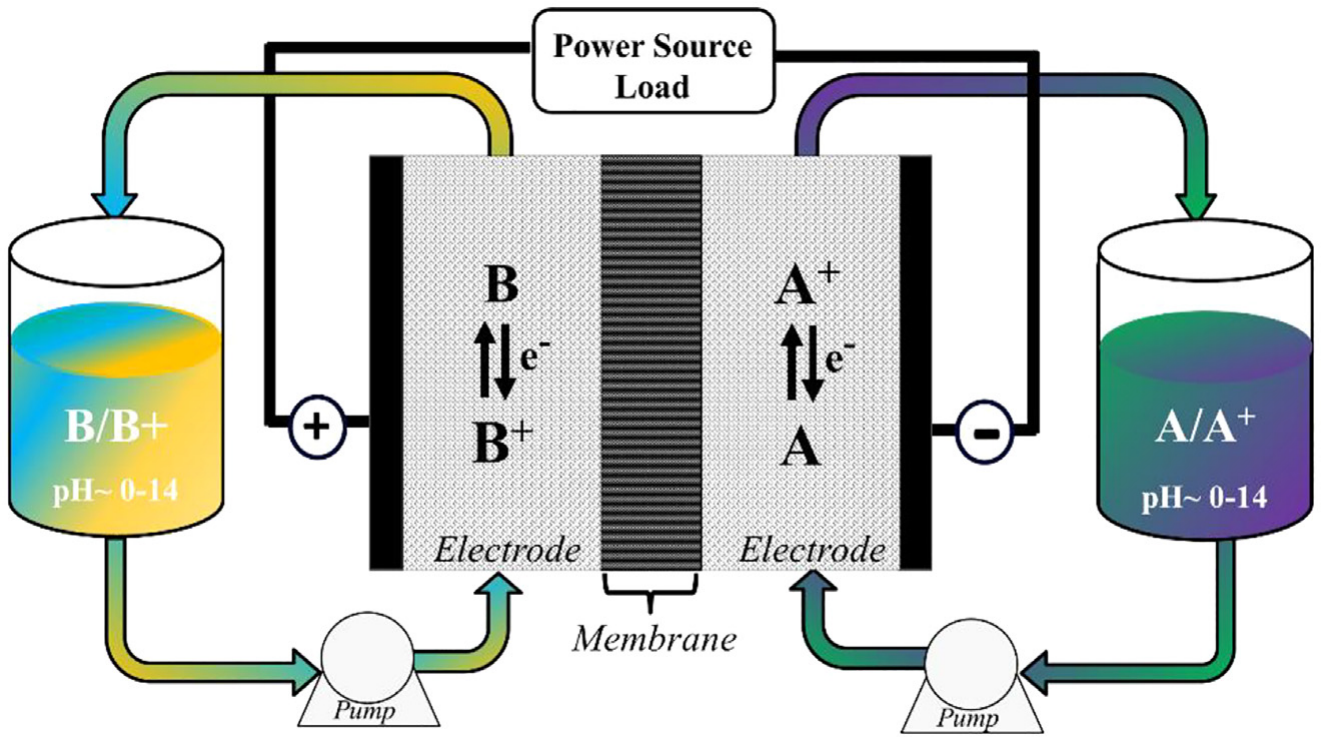
Schematic of a redox flow battery using redox species of A/A^+^ and B/B^+^ on the negative and positive sides, respectively

An RFB system converts chemical energy into electrical energy, and generally employs metal-based redox species dissolved in liquids, which are stored in tanks and then pumped through an electrochemical cell at charge and discharge (Figure 2). Various chemistries can be used for RFBs, and several systems have so far been scaled up from lab- or pilot-scale to commercial scale, such as all-vanadium and zinc-bromine RFBs. These systems, particularly all-vanadium RFBs, meet sustainability criteria mainly due to their long lifespan and the possibility of using electrolytes indefinitely. However, vanadium is scarce and on the list of critical materials. The sustainability of RFBs thus needs to be improved.

**Figure 3:**
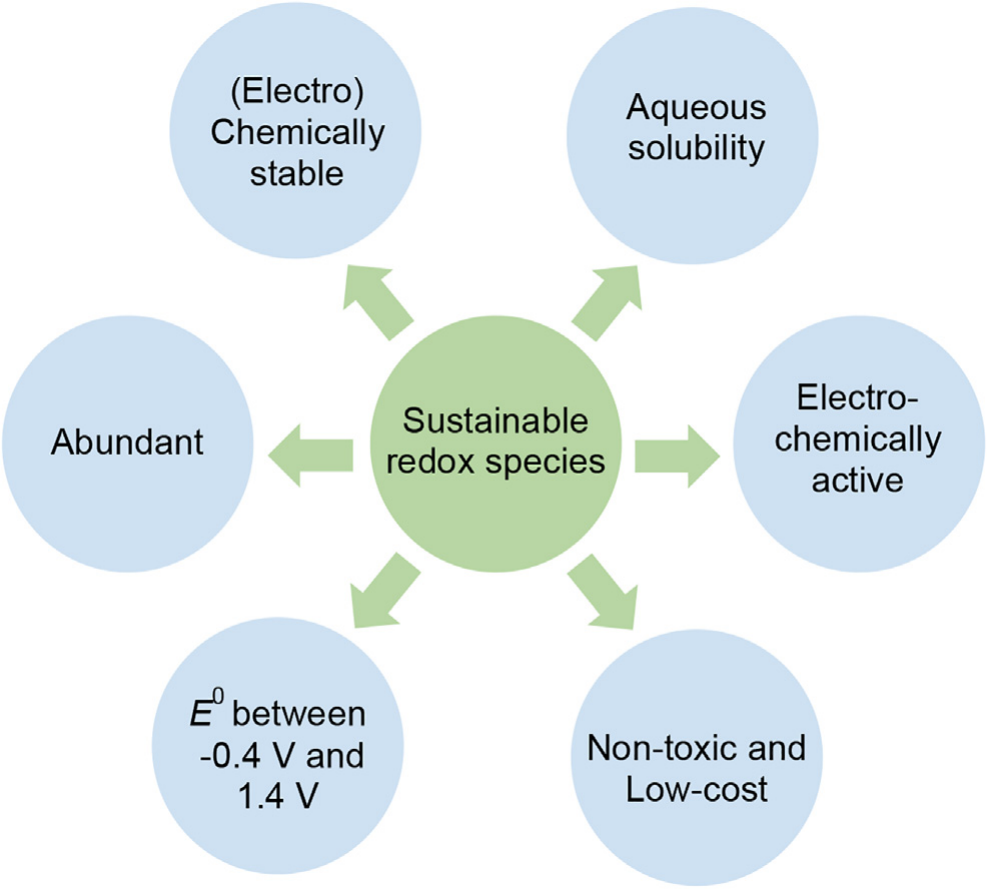
Characteristics of ideal redox species

One of the parameters that contributes significantly to the sustainability of RFBs is the electrolyte. Although all-vanadium RFBs are classified as sustainable, the capital costs ($400–800 kWh^−1^) are far from the target cost for electrochemical energy storage devices ($150 kWh^−1^),^60^ mainly due to the high cost of vanadium pentoxide.^61^ The sustainable redox species (A/A^+^, B/B^+^) used in RFB electrolytes should ideally have the characteristics presented in Figure 3.

Organic redox species have recently attracted attention in an attempt to reduce capital costs and develop green RFB systems. The quinone family is a good example of suitable organic redox species, with an average cost of $5/kg, which is one-sixth of the price of vanadium ($30/kg), the most common metal-based redox species.^26^^,^^62^ It should be mentioned here that there are potential low-cost metal-based redox species such as iron; however, the complexity of the electrochemical reaction and sensitivity to slight changes in pH constitute problems in the upscaling of iron-iron RFBs. Moreover, organic redox species are naturally abundant and can be extracted from various natural sources,^63^ and their electrochemical properties and solubility can be tuned by the pH of the electrolyte and by introducing functional groups into the chemical structure.^26^

Various organic redox species have been developed for RFB systems, such as quinones, anthraquinones, heterocycles, and (2,2,6,6-tetramethylpiperidin-1-yl)oxyl (TEMPO) derivatives. The redox species can be used on the positive or negative side based on their standard redox potential. For instance, a total organic RFB based on anthraquinone-2-sulfonate on the negative side and 1,2-benzoquinone-3,5-disulfonic acid on the positive side demonstrated high coulombic efficiency.^64^ Wang et al. paired 2,2,6,6-tetramethyl piperidine-1-oxyl (positive side) with methyl viologen (negative side) at neutral pH.^65^ The system showed a cell potential as high as an all-vanadium system, and a stable discharge capacity over more than 100 cycles.^66^

Aziz et al. have reported a stable aqueous semi-organic RFB using acidic solutions of 9,10-anthraquinone-2,7-disulfonic acid and Br_2_/Br^−^ redox species.^67^^,^^68^ The system exhibited stable performance with a coulombic efficiency of >99% and capacity retention of 99.84%. In another study, the cell potential was increased from 0.9 V to 1.3 V using a pH differential system.^69^ An alkaline semi-organic RFB with a cell potential of 1.2 V has also been studied using 2,6-dihydroxyanthraquinone and ferrocyanide–ferricyanide as redox species.^70^ The system exhibited 100 stable cycles with a maximum power density of 0.7 W/cm^2^.

Lignin-based electrolytes are also promising candidates for RFBs that fulfill the requirements for sustainability. The two main lignin extracts, kraft lignin and lignosulfonates, meet most of the ideal redox species requirements for RFBs shown in Figure 3. They are both abundant, at an extremely low cost (kraft lignin, $380/MT and lignosulfonates, $340/MT),^1^ and are soluble in aqueous solution.^71^

**Scheme 3:**
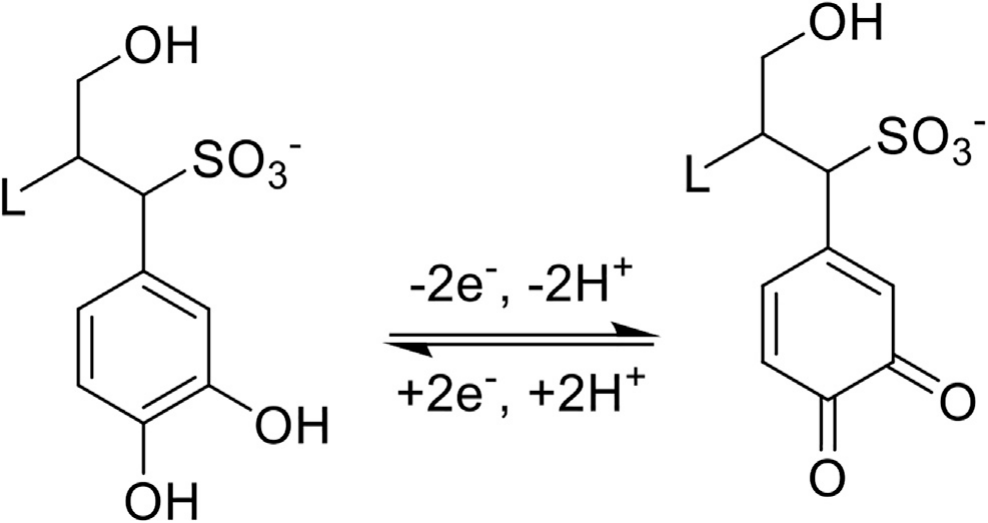
Redox reaction of a representative lignin molecule with a phenol end involving two electrons and two protons^13^

Due to the high content of phenol groups in their chemical structure, kraft lignin and lignosulfonates have excellent potential to quinone/hydroquinone redox reactions.^72^^,^^73^ More precisely, the three main monolignols in the lignin structure, namely *p*-coumaryl, coniferyl, and sinapyl alcohols, contain at least one hydroxybenzene group.^71^ Mukhopadhyay et al. reported the electrochemical reaction mechanism of ultrafiltered lignosulfonate electrolytes in acidic media.^74^ Cyclic voltammetry tests were performed to investigate the electrochemical behavior of the electrolyte, and it was found that electrochemical reversibility is highly dependent on the electrode material and scan rate. The best reversibility was achieved on carbon paper at a scan rate of 60 mV/s and a standard potential of ∼770 mV vs. SHE (standard hydrogen electrode). The electrochemical reversibility to the redox reaction of quinone and hydroquinone is correlated (Scheme 3).

A semi-organic RFB was tested by pairing acidic lignosulfonate electrolytes on the negative side with a bromine solution on the positive side. The RFB was tested at a maximum current density of 20 mA cm^−2^ at a cell resistance of 15 Ω cm^2^. It should be mentioned that the electrochemical reaction mechanism and reversibility of lignosulfonates can be influenced by the pulping liquor concentration, filtration, and the pH of the electrolyte.^71^ However, in the case of electrochemical irreversibility, it is still feasible to obtain quinones or benzoquinones by oxidative depolymerization of lignosulfonates. For example, Areskogh and Henriksson treated commercial-grade lignosulfonates with Fe (II) acetate and H_2_O_2_ under acidic and alkaline conditions.^75^ The results showed that when no Fe (II) acetate was used, more phenolic hydroxyls were formed compared to carboxylic acid, probably through the action of cationic and anionic oxidation species of H_2_^+^OOH and ^−^OOH in acidic and alkaline media, respectively.

In a similar study on alkaline Fenton chemistry, Hocking and Crow reported the depolymerization of the lignin model compound, *p*-hydroxyacetophenone, using H_2_O_2_.^76^ They found that the oxidation of *p*-hydroxyacetophenone had the highest rate when 0.3 M sodium carbonate or sodium hydroxide solution was used. Hydroquinone monoacetate and the peracetate ion were generated and consumed during oxidation. The final products following the Baeyer-Villiger type rearrangement were hydroquinone and acetate ion. Furthermore, it was found that *o*-hydroxyacetophenone was depolymerized via the same mechanism, whereas *m*-hydroxyacetophenone did not react with the H_2_O_2_.

Another lignin model compound, vanillin, has been oxidized to 2-methoxyhydroquinone at high yields of 75.8% under an ambient atmosphere using H_2_O_2_ under alkaline conditions.^77^ The 2-methoxyhydroquinone showed a solubility of 140 g L^−1^ in 0.5 M H_3_PO_4_. In addition, excellent reversibility was achieved in cyclic voltammetry tests with a standard potential of ∼515 mV vs. SHE. The RFB system was developed using *p*-benzoquinone and 2-methoxyhydroquinone on the negative and positive sides, respectively. Cycling tests were conducted over 250 cycles with an average coulombic efficiency of 98%.

## Conclusions and outlook

Lignin is an important bio-based carbon source in the development of innovative new materials and technology. In the area of plastics production, lignin shows particularly good potential as a starting material for new aromatic polymers. So far, many interesting academic advances have been made in the development of lignin-based polymers. However, commercialization of lignin-based plastics has not yet been realized. The main challenges include the difficulty in obtaining pure monomers from the complex lignin structure, as well as the relatively high cost of the lignin-based building blocks (compared to the fossil-based counterparts). While the former challenge will require continued exploration through various biotechnological or chemical engineering approaches, the latter challenge underlines the importance of developing lignin-based polymers with enhanced properties that can outcompete the lower-cost fossil-based plastics in conventional applications, or could be suitable for various niche concepts. Finally, the end-of-life aspects of any lignin-based polymer should be considered from the beginning of the molecular design or synthetic approach in order to ensure the recyclability and biodegradability of the new polymer. This is an important societal and industrial requirement, not only for lignin-based plastics, but also for any new plastics in general.

Oxidative depolymerization has been shown to be able to produce quinone-like structures from the lignin macromolecule. However, the viability of these products has not been tested with regard to their use in redox-flow battery applications. Aqueous flow batteries are a promising technology for future energy storage, mainly due to their greater potential for higher capacity and energy efficiency than non-aqueous RFBs. Employing lignin and its model compounds as redox species constitutes a considerable step toward the sustainability of aqueous flow batteries. The lignin molecule is rich in phenols, and provides an excellent starting point for various quinone-like structures. However, the method of treatment remains the main challenge. Depolymerization should be controlled and selective to produce as many monomers and aromatic species as possible, rather than cleaving the carbon–carbon and carbon–oxygen bonds. On the other hand, the electro(chemical) stability of organic redox species during the charge/discharge of RFBs has long been a problem. Oxidation of reduced species under ambient atmospheric conditions or disproportionation of radicals leads to self-discharge and significant capacity loss. Lignin and its model compounds are also prone to the aforementioned mechanisms, and further research is required to overcome such challenges.

## References

[1] Bajwa D.S., Pourhashem G., Ullah A.H., Bajwa S.G. (2019). A concise review of current lignin production, applications, products and their environment impact. Ind. Crops Prod..

[2] Jiang L., Wang C.-G., Chee P.L., Qu C., Fok A.Z., Yong F.H., Ong Z.L., Kai D. (2023). Strategies for lignin depolymerization and reconstruction towards functional polymers. Sustain. Energy Fuels.

[3] Wallberg O., Jönsson A.S., Wimmerstedt R. (2003). Fractionation and concentration of kraft black liquor lignin with ultrafiltration. Desalination.

[4] Liu H., Xu T., Liu K., Zhang M., Liu W., Li H., Du H., Si C. (2021). Lignin-based electrodes for energy storage application. Ind. Crops Prod..

[5] Bamgbopa M.O., Fetyan A., Vagin M., Adelodun A.A. (2022). Towards eco-friendly redox flow batteries with all bio-sourced cell components. J. Energy Storage.

[6] Argyropoulos D., Crestini C., Dahlstrand C., Furusjö E., Gioia C., Jedvert K., Henriksson G., Hulteberg C., Lawoko M., Pierrou C. (2023). Kraft Lignin: A Valuable, Sustainable Resource, Opportunities and Challenges. ChemSusChem.

[7] Sen S., Patil S., Argyropoulos D.S. (2015). Thermal properties of lignin in copolymers, blends, and composites: a review. Green Chem..

[8] Zhou S.-J., Wang H.-M., Xiong S.-J., Sun J.-M., Wang Y.-Y., Yu S., Sun Z., Wen J.-L., Yuan T.-Q. (2021). Technical Lignin Valorization in Biodegradable Polyester-Based Plastics (BPPs). ACS Sustain. Chem. Eng..

[9] Moreno A., Sipponen M.H. (2020). Lignin-based smart materials: a roadmap to processing and synthesis for current and future applications. Mater. Horiz..

[10] Boarino A., Klok H.-A. (2023). Opportunities and Challenges for Lignin Valorization in Food Packaging, Antimicrobial, and Agricultural Applications. Biomacromolecules.

[11] Choi C., Kim S., Kim R., Choi Y., Kim S., Jung H.y., Yang J.H., Kim H.-T. (2017). A review of vanadium electrolytes for vanadium redox flow batteries. Renew. Sustain. Energy Rev..

[12] Brushett F.R., Aziz M.J., Rodby K.E. (2020). On Lifetime and Cost of Redox-Active Organics for Aqueous Flow Batteries. ACS Energy Lett..

[13] Winsberg J., Hagemann T., Janoschka T., Hager M.D., Schubert U.S. (2017). Redox-Flow Batteries: From Metals to Organic Redox-Active Materials. Angew. Chem. Int. Ed. Engl..

[14] Li C., Zhao X., Wang A., Huber G.W., Zhang T. (2015). Catalytic Transformation of Lignin for the Production of Chemicals and Fuels. Chem. Rev..

[15] Abdelaziz O.Y., Clemmensen I., Meier S., Costa C.A.E., Rodrigues A.E., Hulteberg C.P., Riisager A. (2022). On the Oxidative Valorization of Lignin to High-Value Chemicals: A Critical Review of Opportunities and Challenges. ChemSusChem.

[16] Abdelaziz O.Y. (2021). Lignin Conversion to Value-Added Small-Molecule Chemicals: Towards Integrated Forest Biorefineries.

[17] Ahmad M., Taylor C.R., Pink D., Burton K., Eastwood D., Bending G.D., Bugg T.D.H. (2010). Development of novel assays for lignin degradation: comparative analysis of bacterial and fungal lignin degraders. Mol. Biosyst..

[18] Becker J., Wittmann C. (2019). A field of dreams: Lignin valorization into chemicals, materials, fuels, and health-care products. Biotechnol. Adv..

[19] Rodrigues Pinto P.C., Borges da Silva E.A., Rodrigues A.E., Baskar C., Baskar S., Dhillon R.S. (2012). Biomass Conversion: The Interface of Biotechnology, Chemistry and Materials Science.

[20] Fache M., Boutevin B., Caillol S. (2016). Vanillin Production from Lignin and Its Use as a Renewable Chemical. ACS Sustain. Chem. Eng..

[21] Miroshnikov M., Divya K.P., Babu G., Meiyazhagan A., Reddy Arava L.M., Ajayan P.M., John G. (2016). Power from nature: designing green battery materials from electroactive quinone derivatives and organic polymers. J. Mater. Chem. A Mater..

[22] Jyothibasu J.P., Wang R.-H., Tien Y.-C., Kuo C.-C., Lee R.-H. (2022). Lignin-Derived Quinone Redox Moieties for Bio-Based Supercapacitors. Polymers.

[23] Jia R., He C., Li Q., Liu S.-Y., Liao G. (2022). Renewable plant-derived lignin for electrochemical energy systems. Trends Biotechnol..

[24] Bozell J.J., Hoberg J.O., Dimmel D.R. (2000). Heteropolyacid Catalyzed Oxidation of Lignin and Lignin Models to Benzoquinones. J. Wood Chem. Technol..

[25] Milczarek G. (2007). Preparation and Characterization of a Lignin Modified Electrode. Electroanalysis.

[26] Wedege K., Dražević E., Konya D., Bentien A. (2016). Organic Redox Species in Aqueous Flow Batteries: Redox Potentials, Chemical Stability and Solubility. Sci. Rep..

[27] Biannic B., Bozell J.J. (2013). Efficient Cobalt-Catalyzed Oxidative Conversion of Lignin Models to Benzoquinones. Org. Lett..

[28] Subbotina E., Rukkijakan T., Marquez-Medina M.D., Yu X., Johnsson M., Samec J.S.M. (2021). Oxidative cleavage of C–C bonds in lignin. Nat. Chem..

[29] Klein J., Kupec R., Stöckl M., Waldvogel S.R. (2023). Degradation of Lignosulfonate to Vanillic Acid Using Ferrate. Adv. Sustain. Syst..

[30] Koelewijn S.-F., Ruijten D., Trullemans L., Renders T., Van Puyvelde P., Witters H., Sels B.F. (2019). Regioselective synthesis, isomerisation, in vitro oestrogenic activity, and copolymerisation of bisguaiacol F (BGF) isomers. Green Chem..

[31] Hernandez E.D., Bassett A.W., Sadler J.M., La Scala J.J., Stanzione J.F. (2016). Synthesis and Characterization of Bio-based Epoxy Resins Derived from Vanillyl Alcohol. ACS Sustain. Chem. Eng..

[32] Wu X., De bruyn M., Trimmel G., Zangger K., Barta K. (2023). High-Performance Thermoplastics from a Unique Bicyclic Lignin-Derived Diol. ACS Sustain. Chem. Eng..

[33] Mankar S.V., Garcia Gonzalez M.N., Warlin N., Valsange N.G., Rehnberg N., Lundmark S., Jannasch P., Zhang B. (2019). Synthesis, Life Cycle Assessment, and Polymerization of a Vanillin-Based Spirocyclic Diol toward Polyesters with Increased Glass-Transition Temperature. ACS Sustain. Chem. Eng..

[34] Koike T. (2012). Progress in Development of Epoxy Resin Systems Based on Wood Biomass in Japan. Polym. Eng. Sci..

[35] Mankar S.V., Wahlberg J., Warlin N., Valsange N.G., Rehnberg N., Lundmark S., Jannasch P., Zhang B. (2023). Short-Loop Chemical Recycling via Telechelic Polymers for Biobased Polyesters with Spiroacetal Units. ACS Sustain. Chem. Eng..

[36] Subbotina E., Montanari C., Olsén P., Berglund L.A. (2022). Fully bio-based cellulose nanofiber/epoxy composites with both sustainable production and selective matrix deconstruction towards infinite fiber recycling systems. J. Mater. Chem. A Mater..

[37] Kwon J., Kim J., Park S., Khang G., Kang P.M., Lee D. (2013). Inflammation-Responsive Antioxidant Nanoparticles Based on a Polymeric Prodrug of Vanillin. Biomacromolecules.

[38] Llevot A., Grau E., Carlotti S., Grelier S., Cramail H. (2015). Renewable (semi)aromatic polyesters from symmetrical vanillin-based dimers. Polym. Chem..

[39] Savonnet E., Grau E., Grelier S., Defoort B., Cramail H. (2018). Divanillin-Based Epoxy Precursors as DGEBA Substitutes for Biobased Epoxy Thermosets. ACS Sustain. Chem. Eng..

[40] Gang H., Lee D., Choi K.-Y., Kim H.-N., Ryu H., Lee D.-S., Kim B.-G. (2017). Development of High Performance Polyurethane Elastomers Using Vanillin-Based Green Polyol Chain Extender Originating from Lignocellulosic Biomass. ACS Sustain. Chem. Eng..

[41] Fache M., Auvergne R., Boutevin B., Caillol S. (2015). New vanillin-derived diepoxy monomers for the synthesis of biobased thermosets. Eur. Polym. J..

[42] Lange W., Kordsachia O. (1981). Preparation and Properties of Polyesters from Vanillin and Syringaaldehyde A Contribution to Possible Utilization of Hardwood Lignins. Holz als Roh-und. Werkstoff.

[43] Pang C., Zhang J., Wu G., Wang Y., Gao H., Ma J. (2014). Renewable polyesters derived from 10-undecenoic acid and vanillic acid with versatile properties. Polym. Chem..

[44] Enomoto Y., Iwata T. (2020). Synthesis of biphenyl polyesters derived from divanillic acid, and their thermal and mechanical properties. Polymer (Guildf).

[45] Mialon L., Pemba A.G., Miller S.A. (2010). Biorenewable polyethylene terephthalate mimics derived from lignin and acetic acid. Green Chem..

[46] Mialon L., Vanderhenst R., Pemba A.G., Miller S.A. (2011). Polyalkylenehydroxybenzoates (PAHBs): Biorenewable Aromatic/Aliphatic Polyesters from Lignin. Macromol. Rapid Commun..

[47] Zamboulis A., Papadopoulos L., Terzopoulou Z., Bikiaris D.N., Patsiaoura D., Chrissafis K., Gazzano M., Lotti N., Papageorgiou G.Z. (2019). Synthesis, Thermal Properties and Decomposition Mechanism of Poly(Ethylene Vanillate) Polyester. Polymers.

[48] Gioia C., Banella M.B., Marchese P., Vannini M., Colonna M., Celli A. (2016). Advances in the synthesis of bio-based aromatic polyesters: Novel copolymers derived from vanillic acid and ϵ-caprolactone. Polym. Chem..

[49] Vasiljevic T., Harner T. (2021). Bisphenol A and its analogues in outdoor and indoor air: Properties, sources and global levels. Sci. Total Environ..

[50] Peng Y., Nicastro K.H., Epps T.H., Wu C. (2018). Evaluation of Estrogenic Activity of Novel Bisphenol A Alternatives, Four Bioinspired Bisguaiacol F Specimens, by in Vitro Assays. J. Agric. Food Chem..

[51] Ma S., Wei J., Jia Z., Yu T., Yuan W., Li Q., Wang S., You S., Liu R., Zhu J. (2019). Readily recyclable, high-performance thermosetting materials based on a lignin-derived spiro diacetal trigger. J. Mater. Chem. A Mater..

[52] Shen M., Vijjamarri S., Cao H., Solis K., Robertson M.L. (2021). Degradability, thermal stability, and high thermal properties in spiro polycycloacetals partially derived from lignin. Polym. Chem..

[53] Payne J., Jones M.D. (2021). The Chemical Recycling of Polyesters for a Circular Plastics Economy: Challenges and Emerging Opportunities. ChemSusChem.

[54] Fang Z., Nikafshar S., Hegg E.L., Nejad M. (2020). Biobased Divanillin As a Precursor for Formulating Biobased Epoxy Resin. ACS Sustain. Chem. Eng..

[55] Statista (2023). https://www.statista.com/statistics/1245249/purified-terephthalic-acid-market-volume-worldwide/.

[56] Rocha I.L.D., da Costa Lopes A.M., Ventura S.P.M., Coutinho J.A.P. (2022). Selective Separation of Vanillic Acid from Other Lignin-Derived Monomers Using Centrifugal Partition Chromatography: The Effect of pH. ACS Sustain. Chem. Eng..

[57] Miller S.A. (2013). Sustainable Polymers: Opportunities for the Next Decade. ACS Macro Lett..

[58] Sun Z., Fridrich B., De Santi A., Elangovan S., Barta K. (2018). Bright Side of Lignin Depolymerization: Toward New Platform Chemicals. Chem. Rev..

[59] Nguyen H.T.H., Short G.N., Qi P., Miller S.A. (2017). Copolymerization of lactones and bioaromatics via concurrent ring-opening polymerization/polycondensation. Green Chem..

[60] U.S. Department of Energy (2013).

[61] Khataee A., Pan D., Olsson J.S., Jannasch P., Lindström R.W. (2021). Asymmetric cycling of vanadium redox flow batteries with a poly(arylene piperidinium)-based anion exchange membrane. J. Power Sources.

[62] Minke C., Turek T. (2018). Materials, system designs and modelling approaches in techno-economic assessment of all-vanadium redox flow batteries – A review. J. Power Sources.

[63] Leung P., Shah A.A., Sanz L., Flox C., Morante J.R., Xu Q., Mohamed M.R., Ponce de León C., Walsh F.C. (2017). Recent developments in organic redox flow batteries: A critical review. J. Power Sources.

[64] Yang B., Hoober-Burkhardt L., Wang F., Surya Prakash G.K., Narayanan S.R. (2014). An Inexpensive Aqueous Flow Battery for Large-Scale Electrical Energy Storage Based on Water-Soluble Organic Redox Couples. J. Electrochem. Soc..

[65] Liu T., Wei X., Nie Z., Sprenkle V., Wang W. (2016). A Total Organic Aqueous Redox Flow Battery Employing a Low Cost and Sustainable Methyl Viologen Anolyte and 4-HO-TEMPO Catholyte. Adv. Energy Mater..

[66] Orita A., Verde M.G., Sakai M., Meng Y.S. (2016). The impact of pH on side reactions for aqueous redox flow batteries based on nitroxyl radical compounds. J. Power Sources.

[67] Huskinson B., Marshak M.P., Suh C., Er S., Gerhardt M.R., Galvin C.J., Chen X., Aspuru-Guzik A., Gordon R.G., Aziz M.J. (2014). A metal-free organic-inorganic aqueous flow battery. Nature.

[68] Chen Q., Gerhardt M.R., Hartle L., Aziz M.J. (2016). A Quinone-Bromide Flow Battery with 1 W/cm2 Power Density. J. Electrochem. Soc..

[69] Khataee A., Wedege K., Dražević E., Bentien A. (2017). Differential pH as a method for increasing cell potential in organic aqueous flow batteries. J. Mater. Chem. A.

[70] Lin K., Chen Q., Gerhardt M.R., Tong L., Kim S.B., Eisenach L., Valle A.W., Hardee D., Gordon R.G., Aziz M.J., Marshak M.P. (2015). Alkaline quinone flow battery. Science (1979).

[71] Lange H., Decina S., Crestini C. (2013). Oxidative upgrade of lignin - Recent routes reviewed. Eur. Polym. J..

[72] Quan M., Sanchez D., Wasylkiw M.F., Smith D.K. (2007). Voltammetry of Quinones in Unbuffered Aqueous Solution: Reassessing the Roles of Proton Transfer and Hydrogen Bonding in the Aqueous Electrochemistry of Quinones. J. Am. Chem. Soc..

[73] Son E.J., Kim J.H., Kim K., Park C.B. (2016). Quinone and its derivatives for energy harvesting and storage materials. J. Mater. Chem. A Mater..

[74] Mukhopadhyay A., Hamel J., Katahira R., Zhu H. (2018). Metal-Free Aqueous Flow Battery with Novel Ultrafiltered Lignin as Electrolyte. ACS Sustain. Chem. Eng..

[75] Areskogh D., Henriksson G. (2011). Fenton’s reaction: a simple and versatile method to structurally modify commercial lignosulphonates. Nord. Pulp Paper Res. J..

[76] Hocking M.B., Crow J.P. (1994). On the mechanism of alkaline hydrogen peroxide oxidation of the lignin model p-hydroxyacetophenone. Can. J. Chem..

[77] Schlemmer W., Nothdurft P., Petzold A., Riess G., Frühwirt P., Schmallegger M., Gescheidt-Demner G., Fischer R., Freunberger S.A., Kern W., Spirk S. (2020). 2-Methoxyhydroquinone from Vanillin for Aqueous Redox-Flow Batteries. Angew. Chem. Int. Ed. Engl..

